# *LSL-Kras^G12D^; LSL-Trp53^R172H/+^; Ink4^flox/+^; Ptf1/p48-Cre* mice are an applicable model for locally invasive and metastatic pancreatic cancer

**DOI:** 10.1371/journal.pone.0176844

**Published:** 2017-05-05

**Authors:** Lixiang Ma, Hexige Saiyin

**Affiliations:** 1 Department of Anatomy, Histology & Embryology, Shanghai Medical College, Shanghai, People’s Republic of China; 2 School of Life Sciences, Fudan University, Shanghai, People’s Republic of China; Indiana University School of Medicine, UNITED STATES

## Abstract

Pancreatic cancer (PC) accumulates multiple genetic mutations, including activating *KRAS* mutations and inactivating *TP53*, *SMAD4* and *CDKN2A* mutations, during progression. The combination of mutant *KRAS* with a single inactivating *TP53*, *SMAD4* or *CDKN2A* mutation in genetically engineered mouse models (GEMMs) showed that these mutations exert different synergistic effects in PC. However, the effect of the combination of *TP53*, *CDKN2A* and *KRAS* mutations on the trajectory of PC progression is unknown. Here, we report a GEMM that harbors *KRAS (Kras*^*G12D*^*)*, *TP53 (Trp53*^*R172H/+*^*)*, *CDKN2A (Ink4*^*flox/+*^*)* and *Ptf1/p48-Cre (KPIC)* mutations. Histopathology showed that KPIC mice developed adenocarcinoma that strongly resembled the pathology of human PC, characterized by rich desmoplastic stroma and low microvascularity. The median survival of KPIC mice was longer than that of *LSL-Kras*^*G12D*^*; Ink4*^*flox/flox*^*; Ptf1/p48-Cre* mice *(*KIC) (89 vs 62 days) and shorter than that of *KRAS (Kras*^*G12D*^*)*, *TP53 (Trp53*^*R172H/+*^*)* and *Ptf1/p48-Cre* (KPC) mice. Moreover, the neoplastic cells of KPIC mice were epithelial, highly proliferative tumor cells that exhibited ERK and MAPK pathway activation and high glucose uptake. Isolated neoplastic cells from spontaneous KPIC tumors showed all molecular profiles and cellular behaviors of spontaneous KPIC tumors, including epithelial-mesenchymal transition (EMT) under drug stress as well as tumorigenic, metastatic and invasive abilities in immunocompetent mice. Furthermore, orthotopic and metastatic tumors of KPIC cells almost recapitulated the pathology of spontaneous KPIC tumors. These data show that in addition to spontaneous KPIC tumors, KPIC cells are a valuable tool for preclinical studies of locally invasive and metastatic PC.

## Introduction

PC is a uniformly lethal malignancy with extremely poor outcomes [1]. The progression of PC is a multistep process, and the tumor accumulates different tumorigenic mutations during the different stages [2]. Early PC progression activates *KRAS* mutations and telomere attrition and subsequently inactivates the tumor suppressor genes *p16*^*Ink4a*^, *p14*^*ARF*^, *TP53*, and/or *SMAD4 [2–4]*. Therefore, h*uman PC often h*arbors high frequencies of multiple mutations, including *KRAS (>90%)*, *p16*^*Ink4a*^ *(>60%*), *p53 (>50%*) and SMAD4 (50%) gene mutations [1, 5]. GEMMs not only provide a powerful tool to dissect the roles of these mutations in PC progression but reveal the synergistic effects of these mutations in disease trajectory, providing a model to test chemotherapeutic drugs in preclinical trials [6, 7].

The combinations of oncogenic *Kras*^*G12D*^ with *Trp53*, *Cdkn2a* or *Smad4* mutations successfully delineated the role of these genes in PC and showed that the synergistic effects of inactivating mutations of tumor suppressor genes with *Kras*^*G12D*^ on PC progression depend on the specific inactivating mutation [8–11]. GEMMs that harbor *Kras*^*G12D*^, *Trp53*^*R172H*^ and *Pdx-1Cre* (known as KPC) recapitulated the pathological and metastatic characteristics of human PC and are widely used in preclinical trials [8, 12, 13]; GEMMs that harbor *Pdx1-Cre*, *LSL-Kras*^*G12D*^ *and Ink4a/Arf*^*lox/lox*^ *(known as* KIC) form extremely aggressive PC tumors that result in a median survival of approximately 8 weeks [9]; PC mouse models that harbor *Kras*^*LSL-G12D/+*^,*Dpc4*^*flox/flox*^ and *p48-Cre* (known as KDD) form MCN early on and later develop aggressive, local invasive and widely metastatic PC characterized by a survival period of 8 months [10]. Furthermore, *Pdx1-Cre; LSL-Kras*^*G12D*^*; Ink4a/Arf*^*lox/+*^ mice developed PC that resulted in a mean survival of 34.2 weeks. The deletion of the *TP53* gene (null heterogenic) in *Pdx1-Cre; LSL-Kras*^*G12D*^*; Ink4a/Arf*^*lox/+*^ mice dramatically shortened the mean survival to approximately 20 weeks [11]. Moreover, mutant *p53*, such as *R175H and R273H*, exhibits gain-of-function activities, including enhancing tumor invasion, metastasis and chemotherapy resistance [14, 15]. However, how Trp53R172H, which reportedly elevates H-RAS activity in head and neck squamous cell carcinomas [16] and is a gain-of-function mutation [17], affects the trajectory of *KRAS* and *CDKN2A* mutations in PC has not yet been reported.

Here, we present a novel GEMM that harbors mutations *KRAS*, *Trp53*^*R172H*^ and *p16*^*ink4a*^. These mice developed local aggressive adenocarcinoma that is similar to human PC in terms of pathology. Moreover, isolated neoplastic cells from KPIC tumors maintained the molecular characteristics of the original *in situ* tumor and exhibited tumorigenicity and metastatic potential in immunocompetent C57BL/6 mice in both orthotopic transplantation or intravenous models. These data show that KPIC is a tool to test drug sensitivity in preclinical trials.

## Materials and methods

### Ethics statements

All mice were maintained under standard housing conditions at the MD Anderson Cancer Center (MDACC) animal facilities, and all animal procedures were reviewed and approved by the MDACC Institutional Animal Care and Use Committee.

All mice had free access to sufficient food and clean water and were visited daily to assess the availability of food and water and the growth of the tumor. A maximum of 5 mice were housed per cage, and the veterinarians at the animal facility examined the mice for infection and fighting injuries after surgery. Tumor-bearing mice were euthanized at or before the humane endpoint stage in a CO_2_ chamber. The veterinarians at the animal facility assessed the mice for humane endpoints, which included walking pain, shivering, a loss of appetite or ascites. All KIC, KPIC, and 9 orthotopic mice reached the humane endpoints, and all other mice were sacrificed before the humane endpoints. *LSL-Kras*^*G12D*^*; Ink4*^*flox/+*^mice were provided by Ronald Dephino, and all other mice were purchased from The Jackson Laboratory (Maine. USA).

Freshly surged tissue samples and paraffin sections of human PC were collected from the Pancreatic Surgery Unit of Zhongshan Hospital Affiliated to Fudan University. All the procedures were approved by the Medical Ethics Committee of Zhongshan Hospital of Fudan University.

### Strains and breeding strategies for *LSL-Kras*^*G12D*^*; LSL-Trp53*^*R172H/+*^*; Ink4*^*flox/+*^*; Ptf1/p48-Cre* (KPIC) mice

The mouse strains for breeding KPIC and KIC mice in this work were included the following alleles: *LSL-KRAS*^*G12D*^ mice [18], *LSL-Trp53*^*R172H/+*^ [19], *Ink4a*^*flox/+*^*[20]* and *Ptf1/p48-Cre* mice [21]. To breed *LSL-Kras*^*G12D*^*; LSL-Trp53*^*R172H/+*^*; Ink4*^*flox/+*^*; Ptf1/p48-Cre (KPIC)* mice, we crossed *LSL-Kras*^*G12D*^*; LSL-Trp53*^*R172H/+*^ (KP) mice with *Ink4*^*flox/flox*^*; Ptf1/p48-Cre* (IC) mice. Alternatively, we crossed *LSL-Kras*^*G12D*^*; LSL-Trp53*^*R172H/+*^*; Ink4*^*flox/+*^ (KPI) mice *with Ink4*^*flox/flox*^*; Ptf1/p48-Cr* (IC) mice. Moreover, to obtain KIC mice, we crossed *Ink4*^*flox/flox*^*; Ptf1/p48-Cr* (IC) mice with *LSL-Kras*^*G12D*^*; Ink4*^*flox/flox*^(KI) mice. We obtained 5 KPIC mice from the first breeding cages and 2 KPIC mice from second cages, and 8-10-week-old immunocompetent C57BL/6 (male) mice were used for orthotopic transplantation or intravenous injections. We used standard genotyping primers provided by The Jackson Laboratory (Maine. USA).

### Perfusion of tumor with Lectin-Alexa 633 and 2-NBDG-Alexa 488

Lectin-Alexa 633 and 2-NBDG Alexa 488 were dissolved in 0.01 mM Phosphate Buffered Saline (PBS). KPIC mice were deeply anesthetized with isofluorane, and the Lectin-Alexa 633 (0.5 mg/mL) and 2-NBDG-Alexa 488 (0.5 mg/mL) mixture was slowly injected into the left ventricle of the heart with a TB syringe. Ten minutes after injection, the KPIC mice were sacrificed by cervical spine dislocation, and the tissues were fixed with 4% paraformaldehyde (PFA) for microscopy analyses.

### Immunofluorescent staining and immunohistochemistry

Fresh tumor samples were fixed in freshly prepared 4% PFA overnight and cryoprotected in 30% sucrose/PBS solution. Thick sections and cell immunofluorescent staining were stained and scanned as previously described [22]. The Z-stack scanned images were 3D reconstructed by the Zen 2009 software using a maximum intensity projection.

Tissues were fixed using freshly prepared 4% PFA, and paraffin sections were prepared following routine histology procedures. Briefly, the sections were deparaffinized in xylene and rehydrated in a decreasing ethanol gradient. Endogenous peroxidase activity was blocked by 3% H_2_O_2_ for 10 minutes at room temperature. Heat-mediated antigen retrieval was performed by placing the slides in a sodium citrate buffer (pH 6.0) and heating them twice in a microwave. Next, the sections were blocked in protein blocking agent (DAKO, USA) at 37℃ for 30 minutes. The sections were then incubated with primary antibodies at 4℃ overnight, washed in PBS, incubated with corresponding HRP-labeled secondary antibodies for 30 minutes, and again washed in PBS. Immunostaining was achieved using 3,3’-diaminobenzidine tetrahydrochloride (DAB/H_2_O_2_). All tissue sections were counterstained with Hematoxylin. The following antibodies were used in immunohistochemistry and immunofluorescent staining: Sox-9 (rabbit polyclonal antibody; Abcam), p53 (rabbit polyclonal antibody; Santa Cruz), Phospho-p44/42 MAPK (Erk1/2) (Thr202/Tyr204) (rabbit polyclonal antibody; Cell signaling), CD34 (rabbit polyclonal antibody; Abcam), Her2/Erb (rabbit polyclonal antibody; Gene Tech), Ki67 (rabbit polyclonal antibody; Abcam), pAkt (ser 473) (rabbit polyclonal antibody; Abcam), E-cadherin (mouse monoclonal antibody; Abcam), pan-Cytokeratin (rabbit polyclonal antibody; Abcam), Vimentin (goat; Sigma), anti-rabbit Alexa Fluor 488 conjugated immunoglobulin G (donkey; Life Technologies), anti-mouse Alexa Fluor 555 conjugated immunoglobulin G (donkey; Life Technologies), anti-goat Alexa Fluor 647 conjugated immunoglobulin G (donkey; Life Technologies), and DAPI (Sigma).

### Isolation of KPIC cells from spontaneous KPIC tumors

After euthanizing KPIC tumor-bearing mice in a CO_2_ chamber at the humane endpoints, tumor tissues were removed and washed with sterilized PBS to remove blood clots and then transferred to cell culture dishes containing cell culture medium (DMEM + 10% bovine serum + 2% antibiotics). The tissues were sectioned into small pieces with a scalpel and plated in 10-cm dishes with limited media to ensure tissue attachment. After a sufficient number of cells had extravasated the tissues, the cells were digested and passaged. We observed that early-passage cells consisted of two cell types: After the 3rd passage, we obtained a cell line with epithelial characteristics. After 30 passages, the cell morphology and growth were stable.

### Chemotherapeutic drug treatment of cells

We treated KPIC and KPC cells with gemcitabine and Abraxane *in vitro*. Gemcitabine was dissolved in sterilized PBS to 100mg/mL, and Abraxane was suspended in Sodium Chloride Injection to 100mg/mL. Briefly, 10×10^4^ cells were plated in each well of a 24-well plate containing coverslips and allowed to incubate overnight before being treated with different doses of chemotherapeutic drugs (5 nm/mL-80 nm/mL). After 36 hours, the coverslips were fixed with 4% PFA for immunostaining.

### Testing the tumorigenicity and metastatic potential of KPIC cells

Male C57BL/6 mice aged 2–3 months were deeply anesthetized with isoflurane, and KPIC cells (passage 3 and 6) suspended in sterilized PBS buffer were then injected into the pancreas using an insulin syringe, as previously described [23]. The pancreas of each mouse (n = 9) was injected 0.5 million cells. Moreover, KPIC cells were slowly injected orbital veins after deeply anesthetizing the mice with isoflurane; each mouse was injected 0.1 ml PBS containing 0.2 million cells. After surgery, the mice were placed on a warming pad until waking and returned to the cage after they began to walk freely. All mice had free access to food and water after surgery and were examined daily by veterinarians for infection and bleeding. All mice were sacrificed at or before the human endpoint stage in a CO_2_ chamber.

### Statistical Analysis

The data were statistically analyzed in GraphPad Prism 5 or SPSS 20. Differences between two groups were analyzed with Student’s *t*-test, and overall survival was estimated with the Kaplan-Meier method. All data are presented as the mean ± s.e.m unless otherwise specified.

## Results

### KPIC mice developed local aggressive pancreatic ductal adenocarcinoma that resulted in a median survival of 3 months

**Fig 1:**
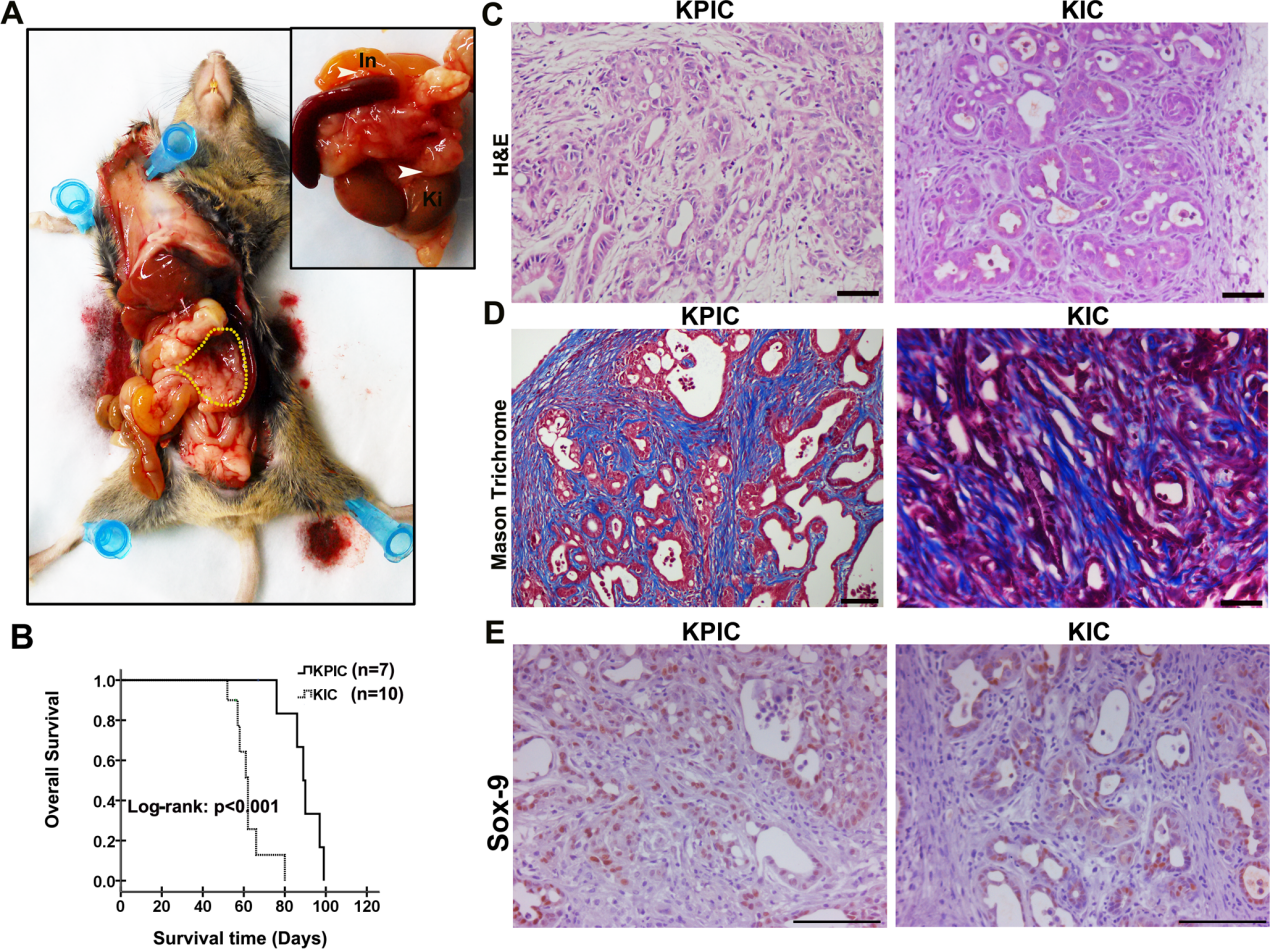
KPIC mice formed a local invasive tumor with 3-months mean survival. (A) The gross anatomy of PC in a KPIC mouse showed cancer in the tail and body of pancreas, and the swelling of small intestine. The KPIC tumor locally invaded the neighboring organs (In, intestine; ki, kidney; yellow circle, tumor region). (B) Kaplan-Merrier curves of KPIC and KIC mice. Log-rank test. (C) H&E and Mason Trichrome staining of KPIC and KIC tumors. Both KPIC and KIC tumors showed a character of typical adenocarcinoma that tumor cells grow in a ductal manner. (D) Mason Trichrome staining showed the dense desmoplastic stroma of KPIC and KIC tumors. (E) Sox-9 immuostaining of KPIC and KIC tumors. Scale bar, 100μm.

We palpated KPIC mice each week starting at 4 weeks to detect tumors. All KPIC mice exhibited palpable hard masses at approximately 7 weeks, and ultrasound scanning confirmed the existence of a hard mass. The tumors of KPIC mice grew quickly thereafter, and KPIC animals developed cachexia, abdominal distension and jaundice at approximately 12 weeks; these characteristics are similar to clinical findings observed in human patients with PC. A gross pathological examination of the anatomy indicated that spontaneous KPIC mice developed locally invasive tumors that infiltrated the spleen, intestine and kidney (Fig 1A). Consistent with clinical syndromes, the swelling of the small bowel, the distension of the gallbladder and hemorrhagic ascites were observed in the abdominal cavities of KPIC mice. These findings are similar to those made in KPC mice [8]. Moreover, in 1 KPIC mouse, the 2 hind legs were completely paralyzed at 13 weeks, and histology revealed a tumor in the spinal cord (Figs not shown). The mean survival of KPIC mice was longer than that of KIC mice (89 vs 62 days) (Fig 1B). Spontaneous KPC and *(KP*^*null*^*IC)* tumors often developed metastatic lesions [8, 11], but we did not observe metastatic lesions in the livers of KPIC mice. The histology showed that 3/5 mice developed typical ductal adenocarcinoma, and 2/5 mice exhibited high-grade panIN; both the adenocarcinoma and high-grade panIN were rich in desmoplastic stroma, which are similar to well-differentiated adenocarcinoma in KIC (Fig 1C and 1D). Human PC of ductal lineage expresses Sox-9 but pancreatic neuroendocrine tumors, acinar cell carcinomas and solid pseudopapillary neoplasms rarely express Sox-9 [24, 25]. To determine if the neoplastic cells in KPIC tumors have the characteristics of ductal lineage, we stained tumor tissue with Sox-9 antibody, which showed that these KPIC tumor cells are positive for Sox-9 staining, as shown in KIC tumor **(Fig 1E).** Taken together, these findings show that spontaneous tumors in KPIC mice exhibit characteristics typical of aggressive human PC.

### The molecular characteristics of KPIC tumor cells are typical of PC

**Fig 2:**
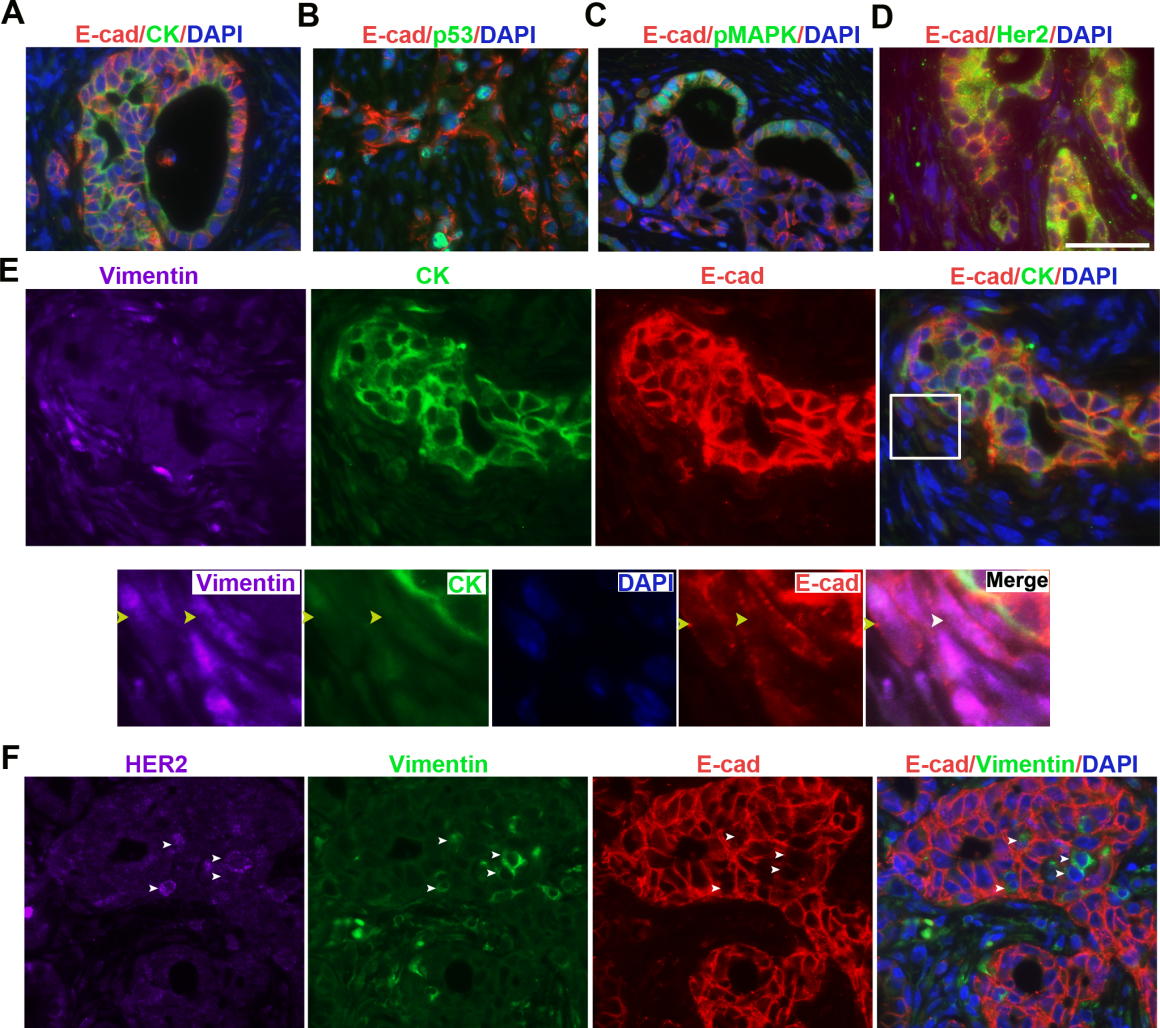
The molecular profiles and EMT of neoplastic cells of KPIC tumors resemble human PC. (A) E-cadherin and CK immunostaining showed that KPIC tumors are epithelial. (B-D) Co-immuostaining p53, pMAPK and Her2 antibodies with E-cadherin antibody in KPIC tumors showed that their molecular profiles are similar to that in human PC. (E, F) Triple immunofluorescent staining of E-cadherin, vimentin and cytokeratin or Her2 staining showed that KPIC tumors harbor a significant number of EMT cells. Her2 expression in EMT cells was upregulated. (circled region, EMT region; white yellow and white arrows, EMT cells). Scale bar, 50μm.

**Fig 3:**
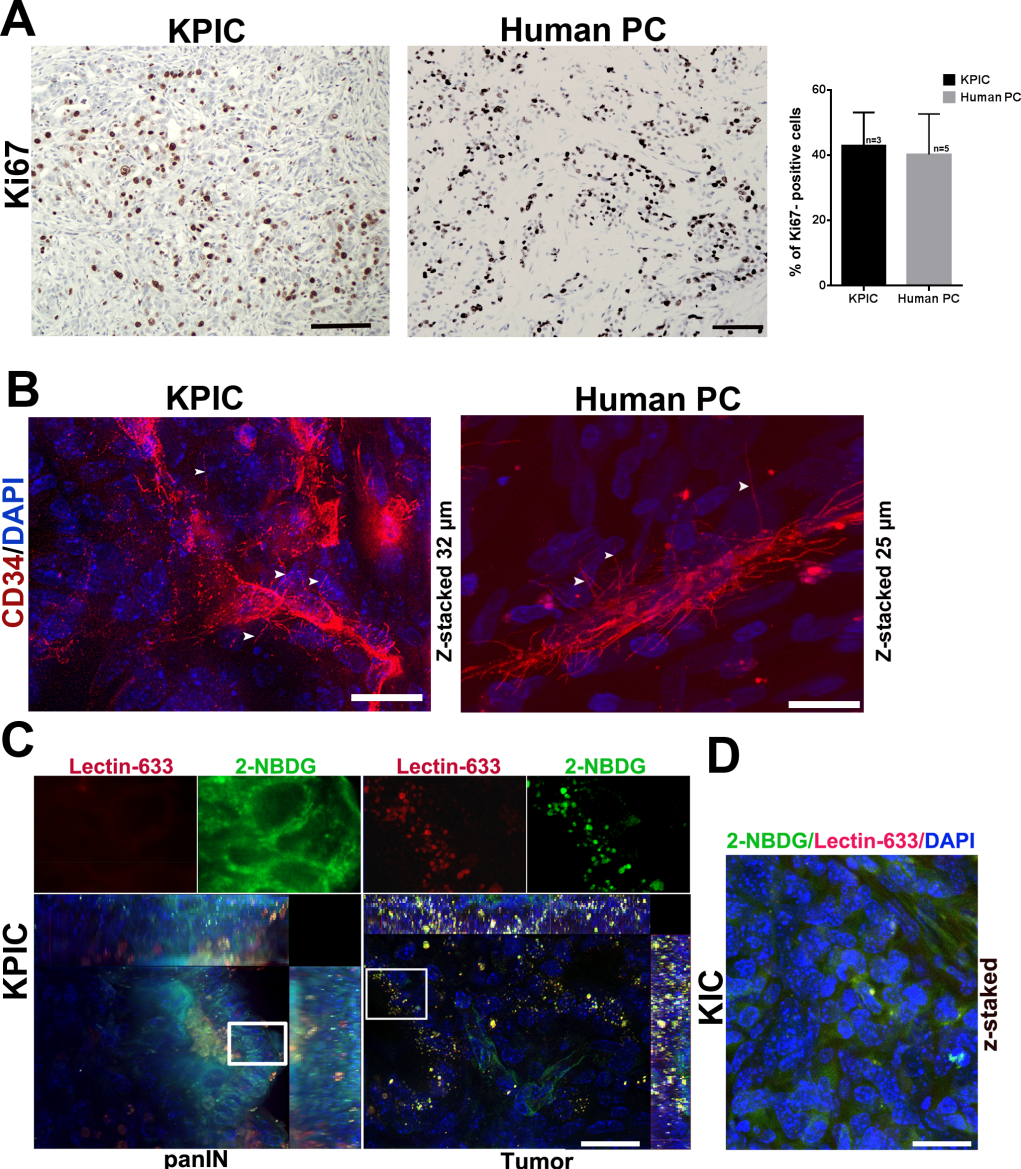
The proliferative and glucose uptake ability and microvasculature characteristics of KPIC tumors. (A) Immuostaining of KPIC tumor and typical human PC with Ki67 antibody showed that the neoplastic cells of KPIC tumor are highly proliferative, and similar to the highly proliferative neoplastic cells in human PC. Scale bar, 50μm. n, sample size. (B) CD34 immunofluorescent staining showed that KPIC tumor has a “hairy” microvasculature with basal microvilli that is rarely branched and similar to the typical “hairy” microvasculature in human PC (Human PC, Age 69, Male, tumor in head of pancreas). Scale bar, 20μm. (C) Accumulations of 2-NBDG-Alex 488 and Lectin-Alex-633 in KPIC tumors. First panel shows a panIN lesion; Second panel shows a typical adenocarcinoma. Scale bar, 20μm. (D) Accumulations of 2-NBDG-Alex 633 and Lectin-Alex 488 in the neoplastic cells of KIC tumors. Scale bar, 20μm.

PC is an epithelial tumor that is positive for E-cadherin and cytokeratin (CK) [26]. To further characterize the neoplastic cells of KPIC tumors, we immunofluorescently double-stained the tumors with E-cadherin and CK antibodies and found that neoplastic cells of KPIC tumors are positive for E-cadherin and CK staining (Fig 2A). Mutant *p53* often causes the abnormal accumulation of P53 protein in neoplastic cells [27]. Moreover, *Kras*^*G12D*^ constitutively activates GTPase, which consequently strongly activates Raf/mitogen-activated protein kinase /mitogen-activated protein kinase (Raf/MEK/ERK) [28]. To assess spontaneous KPIC tumors for the presence of *p53* and *Kras* gene mutations, we measured the expression of p53, phosphorylated (p)MAPK and Her2 in KPIC tumors by double-staining the tumors with E-cadherin antibody combined with p53, pMAPK (Thr202/Tyr204) or Her2/Erb2 antibodies. We found that the neoplastic cells of KPIC tumors expressed high level of p53, pMAPK and Her2 (Fig 2B–2D). Furthermore, *p53* gene mutation in pancreatic cells is known to increase drug resistance and aggressive behaviors [27], and drug resistance and local invasion is often linked to epithelial-to-mesenchymal transition (EMT) in neoplastic cells in PC [29]. The local invasion of KPIC tumors into the surrounding organs suggested that the neoplastic cells in KPIC mice are capable of EMT. To assess the EMT status in KPIC tumors, we triple-immunostained the KPIC tumor with E-cadherin, CK and vimentin antibodies. We found that the neoplastic cells of KPIC tumors contained a few E-cadherin- and vimentin-positive and CK-negative cells (Fig 2E), and these cells were also positive for Her2 (Fig 2F). CDKN2/INK4a binds to cdk4, which acts upstream of Rb to cause G1 arrest [30], and neoplastic cells in *Ink4a*-mutated tumors are highly proliferative [9]. To test the proliferating status of KPIC tumor cells, we stained the tissues with an antibody against Ki67, which is a cell proliferation marker, and found a high percentage of Ki67-positive cells in the neoplastic cells of the KPIC tumor (>50%) (Fig 3A). These data show that KPIC tumors recapitulated the typical molecular profiles, proliferative ability and EMT characteristics of human PC.

Human PDAC and spontaneous KPC tumors are characterized by a rare and "hairy" microvasculature, with poor perfusion and high glucose uptake [22, 31, 32]. To characterize the microvasculature of the KPIC tumor, we stained the microvasculature with CD34 antibodies and found that microvessels rarely branched, and basal microvilli grew on these vessels, as observed in human PC (Fig 3B). To test the glucose uptake ability of KPIC tumors, we injected 2-NBDG-Alexa 488, a fluorescent glucose analog, and Lectin- Alexa 633 into the left ventricles of mice. Surprisingly, the KPIC tumor cell not only accumulated large amounts of 2-NBDG but also fluorescent lectin in tumor cells (Fig 3C). PC cells with *Kras* gene mutation often show a stronger macropincytic ability [33], the accumulations of Lectin-Alexa 633 in the KPIC malignant cells are consistent with a stronger macropinocytic ability of *Kras-* mutated cells. However, we only observed few 2-NBDG and lectin accumulation in KIC tumor cells (Fig 3D). These data show that glucose uptake is higher in KPIC tumors than in KIC tumors and that KPIC tumors better physiologically resemble human PC than KIC tumors.

### Neoplastic cells isolated from KPIC tumors exhibit chromosome instability and rapid proliferation

**Fig 4:**
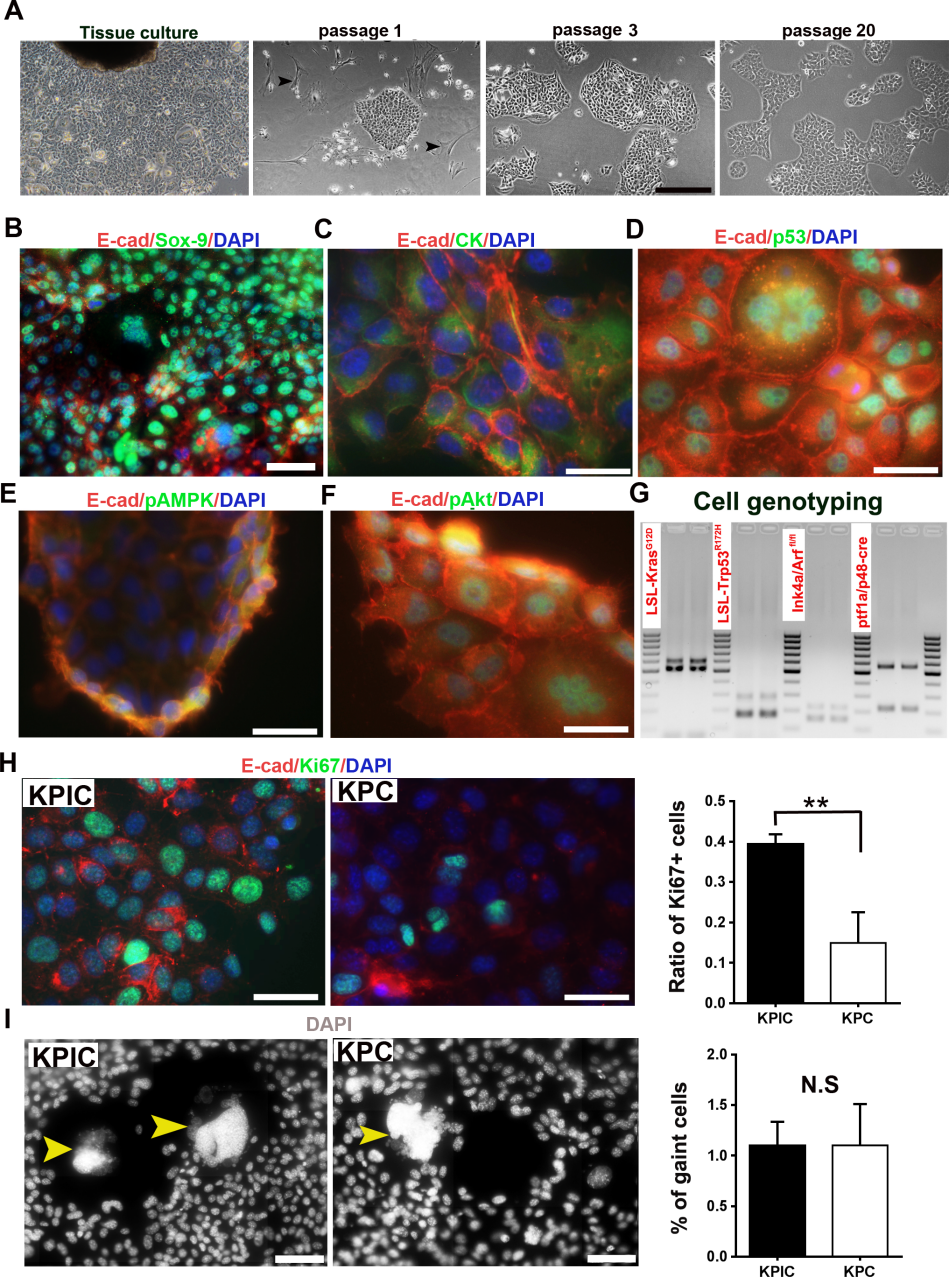
The isolation and characterization of the neoplastic cells of KPIC tumor. (A) The morphology of KPIC cells showed that KPIC cells have the characteristics of epithelial cells, and formed an adhesive, dense and compact monolayer. Scale bar, 100μm. (B-F) Co-immunostainig of KPIC tumor cells with Sox-9, CK, p53p, pMAPK, and pAkt (ser 473) antibodies and E-cadherin antibody. Scale bar, 50μm (B); 20μm (C-F). (G) Genotyping of KPIC cells showed that KPIC cells carried four mutant alleles. (H) Ki67 staining showed that the proliferation of KPIC cells are faster than that of KPC cells. **, P<0.01 using student *t-*test. Scale bar, 50μm. (I) Comparing aneuploid cells in KPIC with KPC cells. N.S, not statistically significant by student *t*-test. Scale bar, 50μm.

To characterize the neoplastic cells of KPIC tumors, we isolated neoplastic cells from spontaneous KPIC tumors. At passage 1, we observed that the isolated cells from KPIC tumor contained stromal cells and epithelial characteristic cells which present an adhesive, dense and compact monolayer pattern (Fig 4A). During passaging, the number of stromal cells were quickly decreased. At passage 3, cells with epithelial morphology were left and stromal cells were almost completely diminished (Fig 4A). The morphology of the isolated cells remains unchanged over 20 passages (Fig 4A). To confirm if the isolated KPIC cells are epithelial, we have co-immunostained the cells with E-cadherin and Sox-9 or CK antibodies. The results showed that the isolated KPIC cells are positive for Sox-9, E-cadherin and CK (Fig 4B and 4C). To see whether the isolated KPIC cells have typical characteristics of cells which harbor *p53* and *Kras* gene mutations, we have co-immunostained the isolated KPIC cells with p53, pMAPK and pAkt antibodies, in combination of E-cadherin antibody. The results showed that the isolated KPIC cells are positive for p53, pMAPK and pAkt. To test whether the isolated KPIC cells carry original four mutated genes, we did a re-genotyping. Genotyping confirmed that cells harbored the original four mutated genes identified in KPIC mice (Fig 4G). Consistent with characteristics of *ink4*-mutated tumors [30], KPIC cells were more proliferative than KPC cells (Fig 4H). *Trp53* mutations often cause the missegregation of the chromosome and multi-nucleated cells, as observed in KPC cells [8]. Consistent with the *Trp53* mutation observed in tumors, 1–2% of both KPIC and KPC cells were aneuploid (Fig 4I). These data showed that the isolated KPIC cells are ductal epithelial cells, and keep the molecular characteristics of *Kras-*, *p53-* and *Ink4-*mutated cells.

### KPIC cells can also undergo EMT in response to gemcitabine and paclitaxel albumin-stabilized nanoparticle (Abraxane) treatment

**Fig 5:**
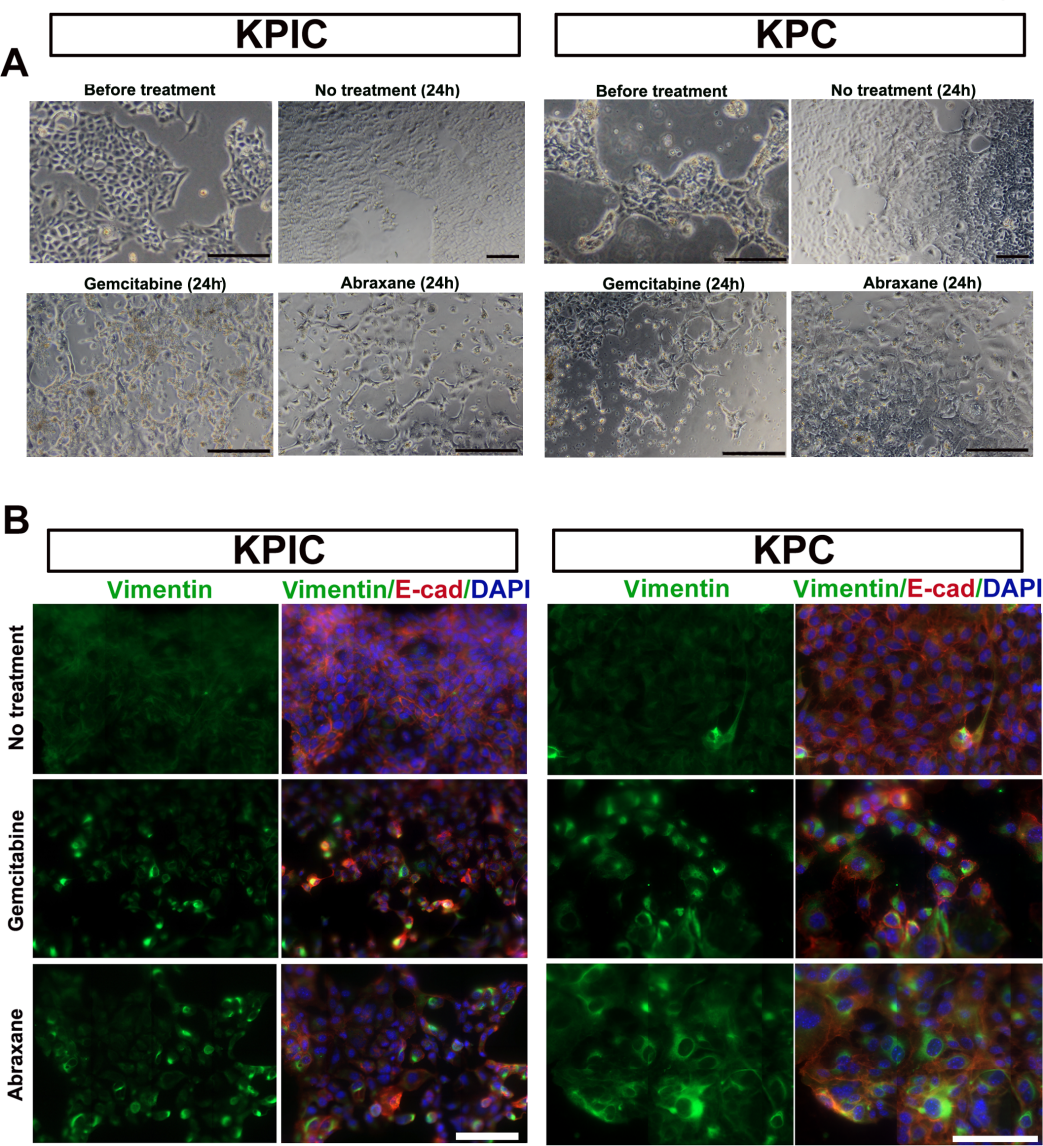
KPIC cells are easy to EMT under drug stress. (A) The morphological changes of KPIC and KPC cells under gemcitabine and Abraxane stresses. (B) Vimentin and E-cadherins staining of gemcitabine and Abraxane treated KPIC and KPC cells. Gemcitabine and Abraxane treatment induced EMT in both cells. Scale bar, 50μm.

*p53* mutation increases the drug resistance of tumor cells [27], and drug resistance is reportedly related to the EMT of epithelial cells in PC tumors [29]. Moreover, paclitaxel- and gemcitabine-resistant epithelial tumor cells often exhibit increased vimentin expression [34–36]. The significant number EMT cells observed in KPIC tissues also implied that these cells easily undergo EMT and are drug resistant. To test the ability of KPIC cells to undergo EMT in response to drug stress, we treated KPIC cells with gemcitabine and Abraxane and observed that both KPIC and KPC cells changed their epithelial morphology to a mesenchymal-like morphology (Fig 5A). Moreover, double-staining with E-cadherin and vimentin antibodies showed that these mesenchymal-like cells expressed vimentin (Fig 5B). These data further show that isolated neoplastic KPIC cells are, like KPC cells, have an ability to EMT under drug stress.

### KPIC cells are tumorigenic and metastatic in immunocompetent mice

**Fig 6:**
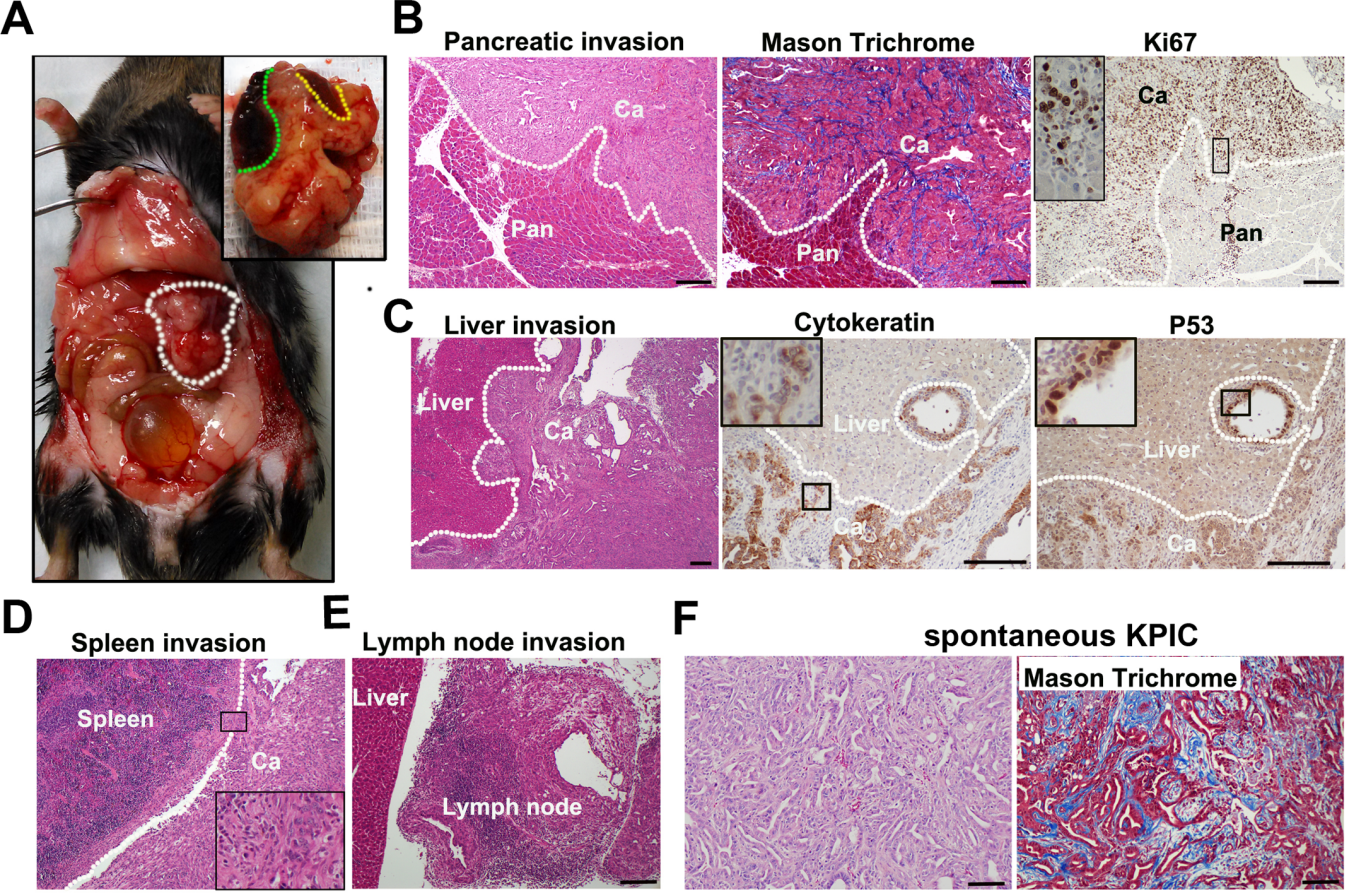
KPIC cells are tumorigenic in immunocompetent mouse. (A) The gross pathology of an orthotopic KPIC cell tumor showed that the orthotopic tumor invaded the spleen and liver (white circle, orthotopic tumor; green line, liver invasion; yellow line, spleen invasion). (B) H&E staining, Mason Trichrome and Ki67 immunostaining of orthotopic KPIC tumors showed that the orthotopic tumors are invasive, and contain desmoplastic stroma and highly proliferative cells. (C) H&E staining, p53 and CK antibodies immunohistochemistry of the orthotopic tumor of KPIC cells showed the invasion of orthotopic KPIC tumor to liver. These KPIC cells are ductal epithelial cells and accumulate p53. (D, E) H&E staining showed the orthotopic tumor invasion to spleen and lymph node. White dotted lines (B-D), the boundary between tumor and normal tissue. Ca, cancer; Pan, pancreas. (F) Histology of original spontaneous tumor of KPIC. Scale bar, 100μm.

Neoplastic KPC cells isolated from spontaneous tumors were tumorigenic in immunocompetent mice [37]. KPC cell orthotopic tumors require 2–4 weeks to be detected, and mice are moribund starting at 4–8 weeks [23, 37]. Thus, KPIC cells should be able to rapidly form tumors in immunocompetent mice. To test the tumorigenicity of KPIC cells in immunocompetent mice (C57BL/6), we orthotopically transplanted KPIC cells to the pancreases of C57BL/6 mice. Consistent with the high proliferative ability of *Ink4a*-mutated cells, we palpated tumor masses in the pancreas starting at 7 days, and these tumors were confirmed by ultrasound scanning. These mice also developed cachexia, abdominal distension and jaundice at approximately 19 days, and most orthotopically transplanted mice became moribund starting at 20 days. A gross pathological examination revealed that KPIC cells also formed locally invasive tumors that resembled spontaneous KPIC tumors (Fig 6A), and these tumors invaded the stomach (1/9), kidney, intestine (9/9) and liver (2/9), which was highly reminiscent of clinical pathological findings observed in humans and in the spontaneous tumor mouse model (Fig 6A). Histology showed that KPIC cells formed an adenocarcinoma that contained a significant amount of stroma and high percentage of Ki67-positive cells, and the growth patterns of tumor cells resembled those of the original spontaneous tumor (Fig 6B–6E). Surprisingly, these tumors also maintained some molecular characteristics of the original spontaneous tumor (Fig 6B–6D).

**Fig 7:**
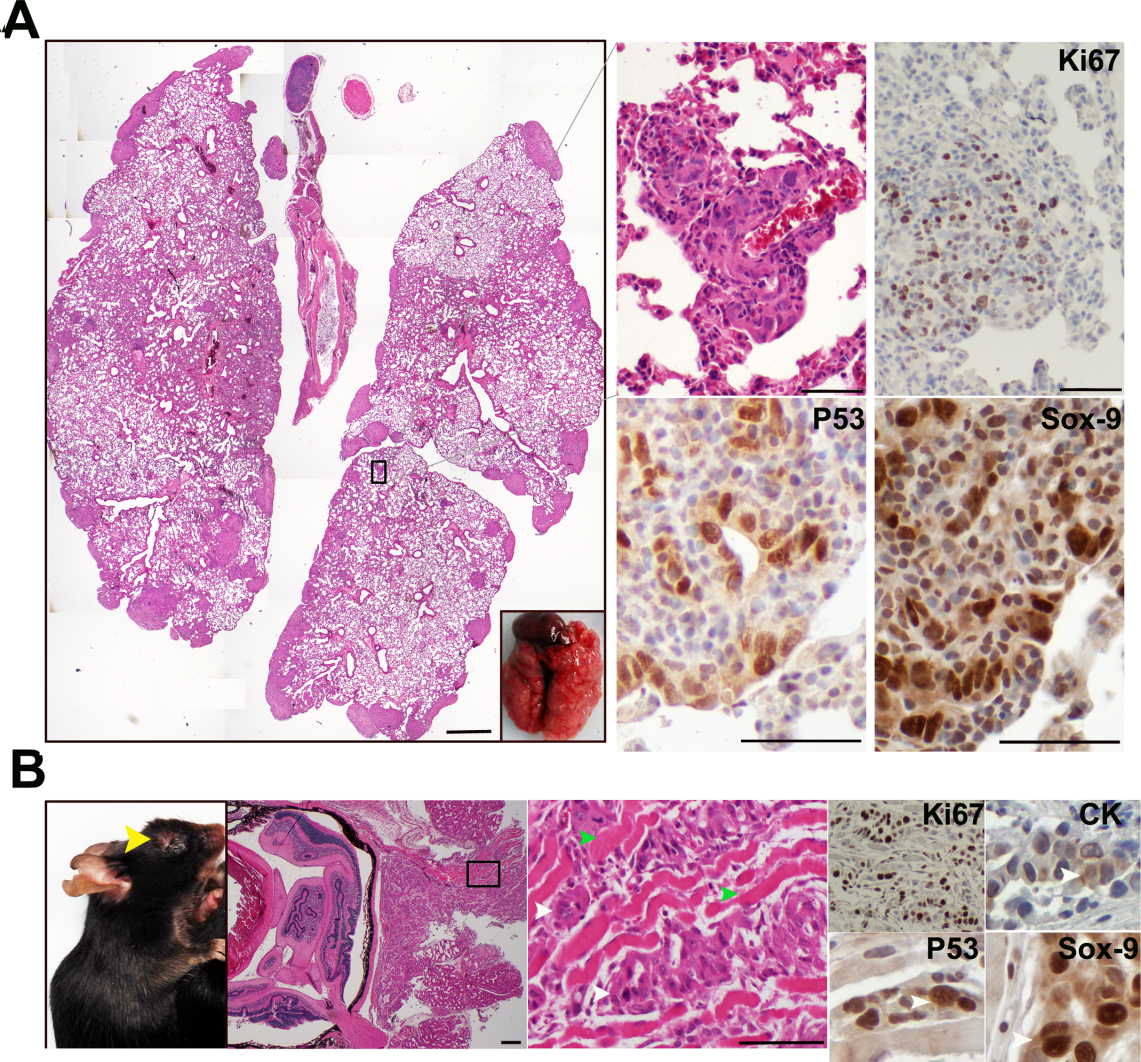
KPIC cells are metastatic in immunocompetent mouse. (A) Orbital intravenous injection of KPIC cells to immunocompetent mice formed multiple metastatic lesions in the lung (19 days). Metastatic KPIC cells are proliferative and Sox-9- and p53-positive. Scale bars, 500μm and 100μm. (B) KPIC cells formed an *in situ* tumor at the injection site which also invaded to the muscles of eye. KPIC tumor cells are proliferative and Sox-9- and p53- positive. (yellow arrow, tumor in eye; green arrows, muscle; white arrows, tumor cells). Scale bar, 100μm.

KPC tumors are highly metastatic, and KPC cells often form metastatic tumors after intravenous injection in immunocompetent mice [8, 38]. Thus, we hypothesized that KPIC cells also have the potential to form a metastatic tumor. To test the metastatic potential of KPIC cells, we injected these cells into the orbital veins of immunocompetent B6/C57 mice, which resulted in the formation of multiple metastatic lesions in the lungs at 2 weeks (Fig 7A); we also observed an invasive *in situ* adenocarcinoma in the eyes of injected mice (2/9) (Fig 7B). These metastatic and *in situ* KPIC tumors also maintain the molecular characteristics of spontaneous tumors, such as rapid proliferation and high p53, Sox-9 and CK expression (Fig 7A and 7B). These data indicated that KPIC cells are also a preclinical model for both invasive and metastatic PC.

## Discussion

PC harbors multiple tumor-driving mutations, including *KRAS*, *P53*, *CDKN2* and *SMAD4* [2, 39]. GEMMs that carry multiple mutations not only expand our understanding of the carcinogenesis of PC but also provide an applicable tool for evaluating novel therapeutic drugs in preclinical trials [2, 7]. To this end, our KPIC mouse models not only recapitulate the pathology and high glucose uptake of human PC but also the molecular characteristics of human patients. In addition, isolated KPIC cells that are orthotopically or intravenously injected into immunocompetent mice are tumorigenic and metastatic, revealing the synergistic effect of mutant *P53* with *KRAS* and *CDKN2*. Thus, these cells are applicable for use in preclinical trials that examine PC with multiple genetic mutations.

Human PC is characterized with rich desmoplastic stroma, a rare and "hairy" microvasculaturity and high glucose uptake ability that relate to drug resistance and poor outcome in PC patients [22,40, 41]. The rich desmoplastic stroma and rare microvascularity in KPIC tumors indicate that the microenvironment of KPIC tumor is similar to human PC and KPC. Moreover, high glucose uptake ability of KPIC tumor resembles the physiological characteristic of aggressive human PC with poor outcome. These data also imply that the chemotherapeutic drug response of KPIC mice might be similar to human PC. Thus, KPIC mice may be used as a model for understanding the mechanism of chemotherapeutic drug resistance in human PC.

Mutant *p53*, including R175H, can increase the invasiveness and metastatic potential of tumor cells [14]. Moreover, KPC and *(KP*^*null*^*IC)* mice develop metastatic tumors, whereas we observed a wide range of local invasion but not metastatic lesions in spontaneous KPIC mice [8, 11], suggesting that *p53* (R172H) might increases the invasiveness, but not metastatic potential of tumors. Furthermore, p16^INK4A^ binds to CDK4 and CDK6 to prevent their conjugation with cyclin D, which inhibits the entrance of cells into the S phase of the cell cycle [42]. Thus, *p16* missense mutations often increase cell proliferation. Therefore, mice may succumb to spontaneous KPIC tumors before developing metastases, which was supported by the formation of metastatic lesions in the lung after intravenous injection and the stronger proliferation of isolated KPIC cells compared with that of KPC cells. Moreover, PC cells with multiple mutations are characterized with EMT under different stresses [29], and neoplastic KPIC cells were capable of EMT in response to stress induced by gemcitabine and Abraxane, which are first-line drugs in the clinic. These data indicate that KPIC cells are an available cellular model for EMT in PC.

KPC mice are the most widely used models for metastatic PC in preclinical trials. However, the median survival of KPC mice is approximately 5 months [8], making this model expensive in terms of both time and money. Alternatively, the KIC model can also be used to study PC [9], but the 2-month survival of KIC mice does not provide a therapeutic window for preclinical trials. Therefore, the approximately 3-month median survival of KPIC mice makes this model economical in terms of time while also might allowing for a short therapeutic window for preclinical trials. Moreover, KPC cells exhibited tumorigenicity in immunocompetent mice [37, 43], and orthotopic transplantation is widely used in preclinical trials. KPIC cells also exhibited tumorigenicity and metastatic potential in immunocompetent mice, and the aggressive patterns of orthotopic KPIC cell tumors resemble those of locally invasive human PC. In addition, KPIC cells formed orthotopic and metastatic tumors in immunocompetent mice faster than KPC cells [43]. Given this rapid formation of locally aggressive and metastatic tumors, KPIC cells are a valuable and economic tool to explore the invasive and metastatic behaviors of PC.

Spontaneous KPIC tumors and KPIC cell orthotopic and metastatic tumors resembled the pathology and physiology of human PC. Thus, the spontaneous KPIC tumors and KPIC cells established in this study provide a valuable tool for understanding the pathology and physiology of PC harboring multiple mutations. Moreover, this model also serves as a tool to test novel therapeutic approaches for PC harboring multiple genetic mutations and to understand the synergistic effects of tumor-driving mutations.
